# Special Issue: Cholinergic Control of Inflammation

**DOI:** 10.3390/ijms23147758

**Published:** 2022-07-14

**Authors:** Roberta Benfante

**Affiliations:** Institute of Neuroscience, Italian National Research Council (CNR), Via Raoul Follereau 3, 20845 Vedano al Lambro, MB, Italy; roberta.benfante@in.cnr.it

Inflammation caused by infection, tissue trauma, and disease states such as arthritis and inflammatory bowel disease is perceived by the Central nervous System (CNS) through different routes that, by means of neural reflex circuits, regulate the immune system response.

Over the last 20 years, the autonomic regulation of local and systemic inflammation through the “cholinergic anti-inflammatory pathway” [1] has been increasingly recognized as playing an important role in therapeutic perspectives in several areas, including neurodegenerative (Alzheimer’s (AD) and Parkinson’s (PD) diseases), psychological and autoimmune (multiple sclerosis (MS)) diseases [2] and metabolic syndrome [3]. Indeed, the deregulation of the immune response, leading to low-grade systemic inflammation, is involved in the etiology of several diseases affecting both the CNS (neuroinflammation) and peripheral organs.

Tracey coined the term “cholinergic anti-inflammatory pathway” (CAIP) [1] following the observations that vagal nerve stimulation attenuated the systemic inflammatory response in rats under endotoxemia, and that the inhibition of pro-inflammatory cytokines was due to the ACh stimulation of nicotinic receptors expressed on macrophages. The afferent arm of the vagus nerve, which contains cytokine receptors, represents the sensory arc of this inflammatory reflex; the efferent arc is represented by the motor arms of the same nerve [4]. At the level of the nucleus tractus solitarius (NTS), vagal afferents stimulated by peripheral inflammatory mediators such as pathogen-associated molecular patterns (PAMP) or pro-inflammatory cytokines activate the cholinergic efferent fibers of the cervical vagus nerve, originating from the dorsal motor nucleus, transmitting the output signals through the celiac ganglion and splenic nerve to the spleen, which is the principal target organ of the inflammatory reflex. Here, a selective population of ChAT^+^ T cells is stimulated to synthesize and release acetylcholine (ACh), which targets both nicotinic (ionotropic, nAChR) and muscarinic (metabotropic, mAChR) acetylcholine receptors (AChR). Experiments with α7nAChR knock-out mice support the importance of the role of this receptor in the CAIP, reviewed in [4], although other nAChRs are present in immune cells. The stimulation of the G protein-coupled mAChR also has effects on the immune system, by contributing to the maintenance of regulatory T cells in antigen-presenting cells in the intestine. In the CNS, acetylcholine signaling through M1 mAChR decreases serum TNFα levels in a mouse model of endotoxemia. At the same time, signaling through mAChRs is associated with pro-inflammatory activities [5]. Moreover, metabolites, nutrients, and intestinal hormones can also stimulate the vagal reflex.

The inflammatory response requires tight control; uncontrolled or excessive inflammation results in unchecked infection or tissue damage and a lethal disease state, such as septic shock. Due to the high impact of inflammatory-based disease on public health worldwide, the high number of reports in the last 20 years highlights the importance of studying the deregulated pathways sustaining inflammation and many reports focused on therapeutic approaches based on pharmacological and electrical modulation by vagus nerve stimulation.

Pharmacological stimulation, as well as inhibition, with cholinergic agonists has shown beneficial results in reducing inflammation in experimental models of neurodegenerative diseases (EAE, PD and AD), and psychiatric conditions (epilepsy, depression, migraine, and schizophrenia) [2]. The controversial results (stimulation vs. inhibition) depended on the experimental model and the type and dose of agonist used.

This Special Issue, “Cholinergic Control of Inflammation”, arises from the need to implement the knowledge on inflammation regulation by the cholinergic system. It is composed of a collection of one original article [6], and five reviews [7,8,9,10,11] that discuss different aspects of how the cholinergic system can respond to or can be affected by inflammatory insults, environmental (acrylamide and pesticides) or infectious (SARS-CoV-2), highlighting possible therapeutic applications. The presence of two commentaries [12,13] enriches the discussion on new intriguing aspects of the involvement of the cholinergic system in response to SARS-CoV-2, again suggesting that the cholinergic system is becoming an increasingly considered target in the pharmacological approach to inflammatory-based diseases.

The increased use of pesticides to control pest and disease in agriculture and livestock has raised the important issue of the impact on non-target organisms as well as humans. In their review, Camacho-Pérez et al. [7] reported that organophosphorus pesticides (OPs) are modulators of cholinergic components, supporting evidence that OPs can influence inflammatory response by deregulating the CAIP. In particular, OPs downregulate the expression and the activity of many cholinergic components (nAChR, mAChR, ChAT, AChE and BuChE, VAChT), thus leading to increased pro-inflammatory cytokine production and decreased anti-inflammatory cytokines production. Since every cell type that express cholinergic components are possible targets of OPs, both acute and chronic exposure to OPs may be related to the development of chronic neurodegenerative disorders, as well as allergies, or immunosuppression phenomena.

Another pollutant that has profound effects on the cholinergic system is acrylamide (ACR). Acrylamide is a contaminant in the most commonly consumed foods, formed during the thermal processing of food. The review by Kopanska et al. [8] summarizes the current state of knowledge on the influence of acrylamide on the cholinergic system and explores the idea that one of the components of ACR toxicity is due to a deregulated inflammatory response. Many scientific reports indicated that the harmful effects of ACR is due to reduced concentration and activity of acetylcholinesterase (AChE), thus leading to an increased level of secreted acetylcholine. In the periphery, an increased level of ACh may lead to CAIP potentiation, but this prolonged activation of α7 can lead to the desensitization of the receptor and in turn to the impairment of the ability to respond to subsequent inflammatory insults, resulting in excessive and chronic inflammation.

The review by Piovesana et al. [9] focuses on the role of α7 nAChR in neuroinflammation and CNS pathologies (AD, MS), discussing possible molecular mechanisms and therapeutic intervention with a new class of α7 agonists, with high selectivity and minimal or no side effects.

The recent pandemic caused by SARS-CoV2, the enormous number of published data focused on COVID-19 pathophysiology and the search for possible therapeutic approaches that could limit the harmful effect of SARS-CoV2 infection also had an impact on the cholinergic system field. Several studies, summarized in the review by Kopanska and collaborators [11], have shown that COVID-19 causes a clear deregulation of the cholinergic system. The discovery that SARS-CoV2 spike protein contains structural and sequence similarities to neurotoxins and agonists capable of binding nAChRs has led the authors to discuss possible implications of the therapeutic potential of these molecules in COVID-19 therapy, along with antiviral drugs, to reduce cytokine production. This review is accompanied by two commentaries [12,13] emphasizing the great debate occurring in the scientific community about this issue.

From the perspective of therapeutic approaches, searching for new molecules with anti-inflammatory activity, the original article by Alberola-Die et al. [6] deals with Peimine, an alkaloid with anti-inflammatory properties, considered one of the main bioactive molecules of *Fritillaria* bulbs (Fb) extracts. Fb has been used as a therapeutic herb for thousands of years, and there is strong support for its beneficial effects and its weak (or lack of) toxicity. So far, the knowledge about its mechanism of action has been limited to the inhibition of voltage-dependent ion channels and muscarinic receptors. The authors present a detailed analysis of Peimine action on the prototype acetylcholine receptor, the muscle-type nAChR, showing for the first time that Peimine is a powerful modulator of nAChR, even at submicromolar concentrations, much lower than the concentration required to block voltage-dependent potassium channels, and muscarinic metabotropic receptors. Docking simulations predicted multiple sites of interactions with nAChR, including the transmembrane domain, and they show that the inhibition of nAChR can occur through several mechanisms that include open-channel blockade and enhanced desensitization. Although it remains to be demonstrated that Peimine regulates other nAChRs, including α7, they suggest that its anti-inflammatory effect might be due to the blocking of α7, an important step in the resolution of inflammation. However, they do not exclude that alternative bioactive compounds from Fb may account for its anti-inflammatory actions, as flavonoids that have already been demonstrated to be positive allosteric modulators of α7, and their enhancement of α7 activity has been proposed as a therapeutic strategies for inflammatory disorders.

Most of the studies on the CAIP derived primarily from research using animal models (e.g., mice). Anatomical and functional differences of the immune system and the autonomic nervous system exist between species. Indeed, many clinical trials in humans failed. One of these differences lies in the emergence of human-specific genes. In the case of the cholinergic system, *CHRFAM7A*—originating from a partial duplication of the α7 nAChR encoding gene, *CHRNA7*—has negative modulatory activity with regard to α7 nAChR function. A detailed description of this gene, the structure of the encoded protein and its role in neurological and inflammatory disease where α7 play a role are discussed in the review by Di Lascio et al. [10]. The authors present evidence that the presence of *CHRFAM7A* may encounter a translational gap to humans, thus suggesting that research with humans is a required step in the developing of successful therapeutic approaches where α7 is the main target.

## Data Availability

Not applicable.
